# Biological Activities Related to Plant Protection and Environmental Effects of Coumarin Derivatives: QSAR and Molecular Docking Studies

**DOI:** 10.3390/ijms22147283

**Published:** 2021-07-06

**Authors:** Vesna Rastija, Karolina Vrandečić, Jasenka Ćosić, Ivana Majić, Gabriella Kanižai Šarić, Dejan Agić, Maja Karnaš, Melita Lončarić, Maja Molnar

**Affiliations:** 1 Faculty of Agrobiotechnical Sciences Osijek, Josip Juraj Strossmayer University of Osijek, Vladimira Preloga 1, 31000 Osijek, Croatia; karolina.vrandecic@fazos.hr (K.V.); jasenka.cosic@fazos.hr (J.Ć.); ivana.majic@fazos.hr (I.M.); gabriella.kanizai@fazos.hr (G.K.Š.); dagic@fazos.hr (D.A.); maja.karnas@fazos.hr (M.K.); 2 Faculty of Food Technology Osijek, Josip Juraj Strossmayer University of Osijek, Franje Kuhača 18, 31000 Osijek, Croatia; melita.loncaric@ptfos.hr (M.L.); maja.molnar@ptfos.hr (M.M.)

**Keywords:** coumarin derivatives, plant protection, antifungal activity, antibacterial activity, nematicidal activity, QSAR, molecular docking, toxicity

## Abstract

The aim was to study the inhibitory effects of coumarin derivatives on the plant pathogenic fungi, as well as beneficial bacteria and nematodes. The antifungal assay was performed on four cultures of phytopathogenic fungi by measuring the radial growth of the fungal colonies. Antibacterial activity was determined by the broth microdilution method performed on two beneficial soil organisms. Nematicidal activity was tested on two entomopathogenic nematodes. The quantitative structure-activity relationship (QSAR) model was generated by genetic algorithm, and toxicity was estimated by T.E.S.T. software. The mode of inhibition of enzymes related to the antifungal activity is elucidated by molecular docking. Coumarin derivatives were most effective against *Macrophomina phaseolina* and *Sclerotinia sclerotiorum*, but were not harmful against beneficial nematodes and bacteria. A predictive QSAR model was obtained for the activity against *M. phaseolina* (*R*^2^*_tr_* = 0.78; *R*^2^*_ext_* = 0.67; *Q*^2^*_loo_* = 0.67). A QSAR study showed that multiple electron-withdrawal groups, especially at position C-3, enhanced activities against *M. phaseolina*, while the hydrophobic benzoyl group at the pyrone ring, and –Br, –OH, –OCH_3_, at the benzene ring, may increase inhibition of *S. sclerotiourum*. Tested compounds possibly act inhibitory against plant wall-degrading enzymes, proteinase K. Coumarin derivatives are the potentially active ingredient of environmentally friendly plant-protection products.

## 1. Introduction

Plant pests and diseases are responsible for major economic losses in agricultural production worldwide. The control of fungal pathogens, pests, and weeds is crucial for crop farming by ensuring efficiency, productivity, quality, and variety of crops [1].

*Sclerotinia sclerotiorum* (Lib.) de Bary, *Macrophomina phaseolina* (Tassi) Goid. and *Fusarium culmorum* (Wm. G. Sm.) Sacc. are nonspecific, polyphagous seed and soil-borne ascomycete fungi [2,3]. *S. sclerotiorum* and *M. phaseolina* infect more than 500 plant species, including oilseed crops, sugar beet, tobacco and vegetables, while *F. culmorum* is among the most destructive Fusarium species and has a wide range of hosts, such as corn, sorghum, small-grain cereals and many wild and tame grass species [4,5]. *Fusarium oxysporum* f. sp. *lycopersici* Snyder & Hansen is a soil-borne, xylem-colonizing ascomycete pathogen of tomato which belongs to the *Fusarium oxysporum* Schltdl. species complex [6]. All of these plant pathogens are widely distributed around the world and cause economically important diseases and reduce yield quality and quantity. *S. sclerotiorum* and *F. culmorum* can cause severe disease symptoms in cooler and wetter areas, while *M. phaseolina* and *F. oxysporum* f. sp. *lycopresici* are particulary severe in areas with a warm climate. They can survive for a long period in plant debris and soil organic matter as resting structures (sclerotia, microsclerotia, thick-walled chlamydospores) or mycelium.

The present use of plant protection products (PPP) has limited the occurrence and development of plant diseases, pests, and weeds. However, their active ingredients are mainly synthetic compounds, poisonous for humans and fauna, and which may also pollute groundwater and soil. Their resistance to pesticides and their environmental and health hazards indicate an urgent need for the development of the active ingredients of PPPs, characterized as highly specific, environmentally friendly, and toxicologically acceptable [1,7].

Coumarins, secondary plant metabolites and their derivatives, demonstrated a wide range of biological activities on different organisms (invertebrate pests, pathogenic fungus and other microorganisms and weeds), as well as their applications in agriculture as eco-friendly plant protection agents. Several coumarin derivatives have been reported as strong antifungal agents against *Sclerotinia sclerotiorum*, *Botrytis cinerea*, *Colletotrichum gloeosporioides*, *Fusarium oxysporum*, *Valsa mali* and *Moniliophthora perniciosa* [8,9,10]. Coumarins also have antimicrobial potential against phytopathogens: *Ralstonia solanacearum* [11], *Agrobacterium tumefaciens* [12], *Pseudomonas aeruginosa* [13,14]. Nematicidal activity has been proven for several simple coumarins, furocoumarines and dicoumarolums, and their skeletons have been used for the development of new efficient nematicides against plant-parasitic nematodes: *Meloidogyne incognita*, *Ditylenchus destructor*, *Bursaphelenchus xylophilus*, *Bursaphelenchus mucronatus* and *Aphelenchoides besseyi* [15,16]. Scarce information is available concerning the impact of coumarin derivatives, potential plant protection agents, on beneficial populations of soil organisms. Beneficial soil organisms have an irreplaceable and significant role in maintaining soil fertility, and the ideal pesticides should not affect this category of organisms. Entomopathogenic nematodes (EPNs) are naturally occurring, beneficial soil invertebrates, but often they are introduced artificially as a biocontrol agent in insect pest management programs in different cropping systems. They are considered as a model organism in studies of medications against gastrointestinal strongylid parasites of mammals [17]. Synthetic pyrazole-5-carboxamide derivatives showed satisfying nematicidal activity and prospects as a new medication against strongylid parasites of sheep [18]. EPNs share ancestral traits with *Caenorhabditis elegans* [19], so they are also an excellent model to investigate selectivity of pesticides on non-target soil organisms. The advantage of entomopathogenic nematodes is resilience after exposure to pesticides and agrochemicals [20], as they could be tank mixed with other pesticides for synergetic effect, which supports the principles of sustainable agriculture.

A modern strategy for the development of plant protection substances includes computer-aided molecular design (CAMD) as a rational approach used for screening, optimization, and the design of new potent agents in plant protection. In silico techniques, such as Quantitative Structure-Activity Relationships (QSAR) and molecular docking, are playing crucial roles in the design of new plant protection agents with improved activity that may later be synthesized and biologically assayed. QSAR techniques provide insight on relationships between chemical structure and biological activity and present an alternative pathway for the design and development of new molecules with improved activity. Using this relationship, the QSAR model is used to predict the activity of new compounds [21]. Thus, Du et al. [22] developed the linear and nonlinear QSAR models for predicting the fungicidal activities of 100 thiazoline derivatives against rice blast caused by *Magnaporthe grisea*. In addition, Cao et al. [23] developed QSAR models for fungicidal activity against 38 *N*-nitrourea derivatives. The two best QSAR models were used to predict the activity of new inhibitors and guide the further modification of these compounds.

Molecular docking is a molecular modelling technique that is used to describe the interactions between receptor (enzyme, protein) and ligand (molecule). Possible targets for research of the mechanism of action antifungal agents are enzymes responsible for the fungal growth. The enzyme responsible for the synthesis of the sterols, such as ergosterol, necessary for their growth and survival of fungi, is lanosterol 14-demethylase (CYP51). Molecular docking studies of azoles, agrochemical antifungals against CYP51, revealed a plausible binding mode for the active compounds, in which the hydroxyl group binds with a methionine backbone carboxylic group blocking access to the iron catalytic site, providing the platform for the design of the future azole antifungals [24]. The hydroxyl group coumarin antifungal lead compounds bind with a methionine backbone carboxylic group blocking access to the iron catalytic site of CYP51 [25]. Chitin synthase is a promising target for developing fungicides, since chitin is a structural component of the fungal cell wall [26,27]. Thus, *Saccharomyces cerevisiae* chitinase 1 family, 18 plant-type chitinase A1 (AfChiA1) was a target for the development of antifungal PPP [28]. The study revealed the crystal structures of the enzyme in complex with the most potent inhibitor (pdb: 4TXE). Another kind of target for molecular docking are fungal exocellular enzymes, such as cellulolytic, hemicellulolytic, pectolytic and proteolytic enzymes, which are capable of degrading plant cell wall components [29].

Active components of plant protection products must be proven safe for peoples’ health, and effects on animal health and the environment. Before the plant protection products’ registration, they must undergo laboratory testing on animals for short-term and long-term health effects. Moreover, the REACH (Registration, Evaluation, and Authorization of Chemicals) guidelines (of the European Parliament) for animal safety restrict the extensive use of animals in testing. The regulation suggests the QSAR approach to predict the intrinsic properties of chemicals by using various databases, theoretical models, and software applications [30].

The aim of this study was to evaluate the inhibitory effects of recently synthesized coumarin derivatives using environmentally safe “green solvents” [31], on plant pathogenic fungi. Moreover, to validate their environmental impact, the compounds were assessed against the soil-beneficial nematodes and bacteria. Additionally, to screen compounds on several toxicity endpoints, without expensive and time-consuming bioassays, toxicity estimation software, which is based on QSAR methodology, was used. QSAR will elucidate the most important structural characteristics of coumarin derivatives for antifungal activity, and an equation for the prediction of antifungal activities of new, untested compounds with improved activity will be proposed. The binding affinity and interactions with the active sites of enzymes responsible for the fungal growth and the plant cell wall-degrading enzymes were evaluated by molecular docking to determine the mode of action of the most active compounds against fungi.

## 2. Results

### 2.1. Synthesis of Coumarin Derivatives

The synthesis of coumarin derivatives was performed in environmentally safe organic solvents, as we described previously [31]. The structures of the analyzed compounds are presented in Table 1.

### 2.2. Biological Activity Evaluation

#### 2.2.1. Antifungal Activity

The antifungal bioassay results are shown in Table 2. The activities of tested compounds against *Macrophomina phaseolina* varied from 24.80% (**13**) to 83.62% (**23**) compared to control. Fungal growth inhibition was more than 80% for three compounds (**23**, **24,** and **38**). Five compounds stimulated the growth of mycelia of *Sclerotinia sclerotiorum*. On the other hand, compounds **6**, **7**, **8**, **9,** and **14** inhibited mycelial growth by more than 80%. The most effective compounds against *Fusarium oxysporum* f. sp. *lycopersici* were **27**, **28** and **29** (71.60%, 65.53%, and 65.53% respectively). Seventeen compounds stimulated the growth of the mycelia of *F. culmorum*. Compounds **24** and **29** inhibited the growth of *F. culmorum* at 70.19%. The inhibition rate (%) of control, 48 h after inoculation for all tested fungi was 0.

#### 2.2.2. Antibacterial Activity

Data presented in Table 2 displayed the minimum inhibitory concentration of all tested compounds against beneficial soil bacteria *Bacillus mycoides* and *Bradyrhizobium japonicum*. Most compounds did not show an inhibitory effect on bacterial growth, except for compound **5,** which had an inhibitory effect on *B. japonicum* at the concentration 64 μg/mL.

#### 2.2.3. Nematicidal Activity

Nematicidal activities of tested coumarin derivatives (expressed as % of inhibition) are presented in Table 2. Most of the compounds did not exhibit nematicidal activity against two beneficial nematode species *Heterorhabditis bacteriophora* and *Steinernema feltiae*. A. The exception was compound **10**, which was lethal for 56.75% *H. bacteriophora* and 64.00% *S. feltiae* after 48 h, as well as compound **24**, which was lethal for 44.50% and 43.00% population of nematodes, respectively. Compound **34** caused high mortality only for *S. feltiae* (40.25%).

### 2.3. Estimation of Toxicity

Table 3 shows the results of the estimated toxicity obtained by The Toxicity Estimation Software Tool (T.E.S.T.) program [32]. A lethal dose for rats (oral rat LD_50_) is a dose of chemical required to kill half the members of a tested population after oral ingestion. The LD_50_ is expressed as the mg of the chemical per bodyweight of the rat (mg/kg bw). Toxicity on water organisms is presented as the concentration of the test chemical in water in mol/L that causes 50% growth inhibition to *Tetrahymena pyriformis* after 48 h (48 h *Tetrahymena pyriformis* IGC_50_) [33]. Aquatic toxicity of the compound is also presented by concentration in water, which kills half of fathead minnows (*Pimephales promelas*) in 96 h [30]. The Ames mutagenicity test estimates mutagenicity of compound that induces revertant colony growth of *Salmonella typhimurium*. Its results represent the alert for the potential carcinogenicity and/or teratogenicity [34]. Bioaccumulation is a process of absorption of compounds in an organism from the natural environment. The bioaccumulation factor (BAF), is the ratio of the concentration of a chemical in the tissue of an aquatic organism (fish) to its concentration in water (in liters per kilogram of tissue), expressed as logarithmic values [35].

According to the acute systemic toxicity classification based on oral LD_50_ values for rats recommended by the Organization for Economic Co-operation and Development (OECD), five compounds (**3**, **31, 33**, **35**, and **37**), with estimated LD_50_ values in the range of 50–300 mg/kg, are characterized as “toxic” [36].

The three highest estimated oral rat toxicity has three derivatives of coumarin with benzoyl radical at the positions C-3: **3**, **35**, **37** (see Table 2 and Table 3).

The highest aquatic toxicity against *Tetrahymena pyriformis* was also estimated for the compounds with the same structural feature (**3**, **8**, **33**, **35**, **37, 38**), as well as for compounds with 3-acetyl substituents (**16**, **20**, **22**). Potentially highly toxic for the fish, fathead minnows, are also compounds **3**, **8**, **22**, **21**, and **38**. Compounds **8** and **35** also showed the greatest potential for bioaccumulation in aquatic organisms. Unfortunately, compounds **14**, **15**, and **31**, for which short-term toxic effects have not been estimated, are potentially mutagenic, as well as **21**, **35,** and **37**, which have been evaluated as highly toxic.

### 2.4. QSAR and Pharmacophore Mapping for Antifungal Activity

Considering that most of the assessed compounds showed expressed activities against two fungi, *M. phaseolina* and *S*. *sclerotiorum*, these data were used for performing the QSAR study. For each fungus was generated specific QSAR model.

#### 2.4.1. QSAR and Pharmacophore Mapping for Activity against M. phaseolina

The best model obtained is:

log % inhibition = 1.82 + 5.55 *JGI6* − 0.72 *Mor28v* − 0.05 *L2e* (1)

*N*(training set) = 23; *N*(test set) = 9 (**4**, **11**, **13**, **18, 32**, **35**). The compounds of the test set were chosen according to the cluster methods. A dendrogram of a cluster formation for the activity against *M. phaseolina* is presented in the Supplementary File (Figure S1). The six compounds with residuals of more than 0.06 were excluded according to the plot of experimental endpoint vs. residuals from the predictions by model equation (**4**, **11**, **13**, **18**, **32**, **35)**. The variables in Equation (1) are listed in order of relative importance by their standardized regression coefficients. Values of molecular descriptors included in the model are listed in the Supplementary File (Table S1). Statistical parameters of the obtained model are presented in Table 4.

**Figure 1:**
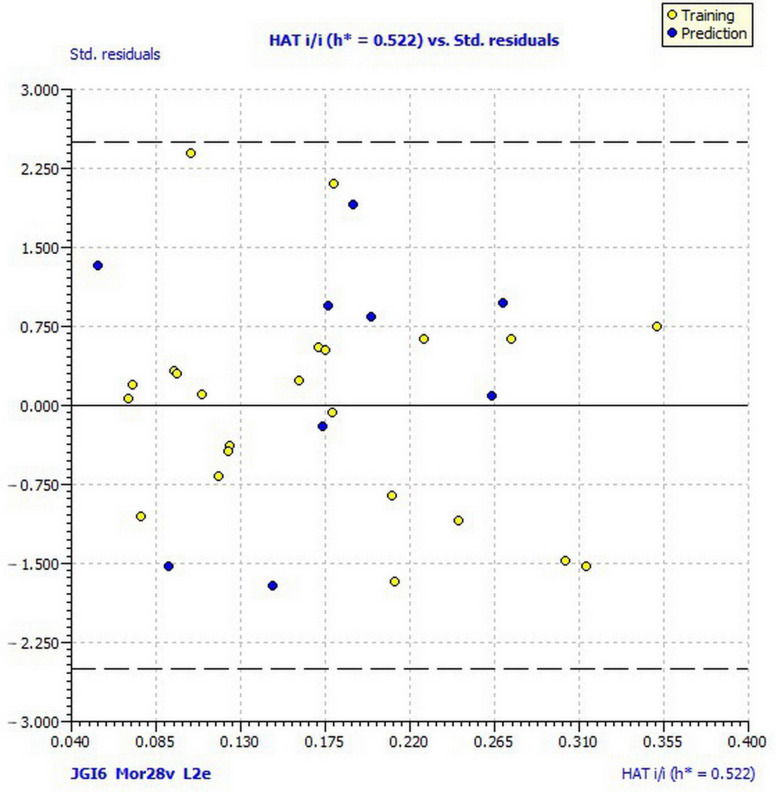
Williams plot of applicability domain of the QSAR model for inhibition of *Macrophomina phaseolina* calculated by model (1).

Experimentally determined activities against *M. phaseolina* with the activities predicted by the best obtained QSAR models and residuals are given in Supplementary File (Table S2). The obtained model satisfied the suggested threshold values of fitting criteria: coefficient of determination (*R*^2^*_tr_*) greater than 0.60, as well as higher or equal to the adjusted coefficient of determination (*R*^2^_adj_). Also, the concordance correlation coefficient of the training set (*CCC*_tr_) is higher than 0.80 [37,38,39]. To test the multicollinearity of the descriptors involved in the model and avoid “apparent” prediction, a power matrix of correlation (1) was generated. Low collinearity was confirmed by the values of the correlation coefficient (*R* ≤ 0.7) and verified with the value of the multivariate correlation index (*Kxx*) and the difference between global correlation among descriptors (Δ*K* ≥ 0.05) [37,40] (Table 4 and Table 5). The stability and robustness of a model were confirmed by parameters of internal validation employing leave-one-out (LOO) cross-validation. According to Chirico and Gramatica [41], the cross-validated squared correlation coefficient (*Q*^2^*_LOO_* ≥ 0.6); the root-mean-square error of the training set determined through the cross-validated method *RMSE_cv_* should be higher than the root-mean-square error of the training set (*RMSE_tr_*) with *MAE_cv_* (mean absolute error of the internal validation set) close to zero. Y-scrambling was performed to eliminate chance correlation. Since the value of Y-scramble correlation coefficient (*R*^2^*_Y_scr__*) and Y-scramble cross-validation coefficients (*Q*^2^*_Y_scr__*) are <0.2, as well as *R*^2^*_Y_scr__* > *Q*^2^*_Y_scr__* (Table 4), and the root-mean-square error of Y-randomization (*RMSE_AV_ Y_scr_*) is higher than *RMSE_cv_*, we can conclude that model (1) was not developed by chance [42]. The values of external validation parameters confirmed the external predictive ability of the model (1) [43,44]. The coefficient of determination of validation set (*R*^2^*_ext_*) is greater than 0.60; concordance correlation coefficient of the test set (*CCC_ext_*) is higher than 0.80, root-mean-square error of the external validation set (*RMSE_ex_*) and mean absolute error of the external validation set (*MAE*_ex_) are close to zero. The coefficient of determination of validation set (*R*^2^*_ext_*), as well as predictive squared correlation coefficients (*Q*^2^*_F_*_1_, *Q*^2^*_F_*_2_, *Q*^2^*_F_*_3_), is higher than 0.60. The average value of squared correlation coefficients between the observed and (leave-one-out) predicted values of the compounds (*r*^2^*_m_ average*) is suitable when the test set size is considerably small, such as in model (1). If its value is higher than 0.50, and its absolute difference less than 0.2, such as in model (1), the closeness between the predicted activity and that of the observed activity is greater [45]. The reliability prediction of the obtained model was defined by the applicability domain. Inspection of Williams plot (Figure 1) revealed that there are no compounds outside of warning leverage (h* = 0.5217) and no outliers, so we can conclude that model (1) can give reliable predictions for chemicals that are similar to those used to develop the model.

The molecular descriptor *JGI6* belongs to the topological charge indices. The descriptor *JGI6* represents the total charge transfer between atoms located at topological distance 6, taking into account the electronegativity of atoms. According to equation (1), molecules with linear substituents (-CN, -OCH_3_, -OC_2_H_5_), and with more heteroatoms, higher values of Pauling electronegativity index can inhibit *M. phaseolina* more successfully [46]. Therefore, the most active molecules (**22**, **23**, **24**, **25**, **38**) with the increased values of *JGI6* (Table 2; Table S2) have substituents with atoms of higher electronegativity (Br, N, O) at the positions C-3 and C-6. The benzoyl group reduces the inhibitory effect of coumarin derivatives.

The second variable in Equation (1) is the 3D-MoRSE descriptor *Mor28v*. The 3D-MoRSE (Molecular Representation of Structures based on Electronic diffraction) descriptor *Mor28v* reflects the contribution of 3D distribution van der Waals volumes at a scattering parameter s = 27 Å^−1^ [47]. This descriptor is extremely sensitive to the position of atoms higher van der Waals volumes (C, Cl, Br). The negative coefficient of the *Mor28v* in the model (1) implies that its higher values correspond to the lower activities. Since interatomic distance participates in the denominator of radial basis function, the smaller distances between atoms higher van der Waals volumes correspond to the lower values of *Mor28v*, therefore the molecule has higher antifungal activity against *M. phaseolina*. Thus, compared to compound **4** (*Mor28v* = 0.075; log % inhibition = 1.752)**,** which has Br atom at the position C-6, compound **38** has one more Br atom at the C-8 position. The presence of an additional Br atom at the position C-8 lowered its value of *Mor28v* (−0.032), which had a positive impact on the inhibition (log % inhibition = 1.904).

The third variable in model (1) is a Weighted Holistic Invariant Molecular (WHIM) descriptor, geometrical descriptors based on statistical indices calculated on the projections of the atoms along principal axes. Descriptor *L2e* is the 2nd component size directional WHIM index, weighted by atomic Sanderson electronegativities. This descriptor represents the variances of the electronegative atoms along with each component, and is also related to the molecular size [48]. Molecules with an increased number of electronegative atoms (O and N) in close contact, such as molecules **14** and **15**, which possess nitro and carboxyl groups, have shown reduced inhibition against pathogenic fungi.

**Figure 2:**
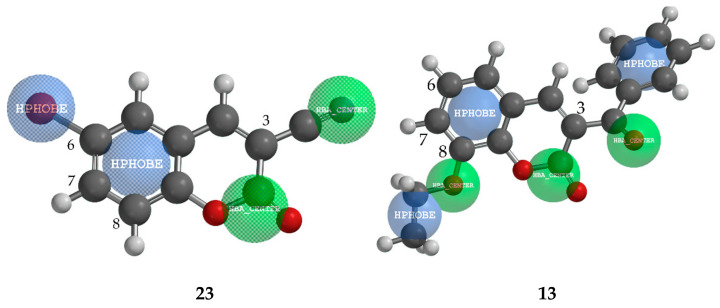
Pharmacophore mapping of the most active compound **23** and of the least active compound **13** against pathogen fungi *M. phaseolina*. (Green = hydrogen-bond acceptor; Blue = hydrophobe region).

According to the results of QSAR analysis, we may conclude that coumarin derivatives with more electron-withdraw groups, such as –Br, –COOR, –COR, and –CN, possess enhanced inhibition effects against *M. phaseolina*, but, the specific position of these groups had a significant impact on antifungal activity. Figure 2 presents a pharmacophore mapping for the most active compound (**23**, 83.62% inhibition), and the least active compound against *M. phaseolina* (**13**, 24% inhibition). Compound **23** at C-3 position possesses easily available hydrogen-bond acceptor, –CN group, and smaller hydrophobic Br atom at the position C-6. In contrast to compound **23**, the least active compound **13** has a large sterically-crowded group, phenyl ring at position C-3, and –OC_2_H_5_ at position C-8, which is possibly unfavourable for the activity because of vicinage hydrophobic –C_2_H_5_ group to the hydrogen-bond acceptors of the pyrone ring.

#### 2.4.2. QSAR and Pharmacophore Mapping for Activity against *S. sclerotiorum*

Ten compounds (**20**, **23**, **30**, **31**, **32**, **33**, **34**, **35**, **37**, **38**) with activities lower than 14% (Table 2), also the members of the first two clusters in the dendrogram (Supplementary File, Figure S2), were excluded from the data set. The limited number of remaining compounds in the data set, (28), did not allow the preliminary splitting of data to training and test set, and statistical external validation.

log % inhibition = 1.53 + 0.19 *SEigm* − 2.43 *P2s* + 2.60 *R1e+* (2)

Values of molecular descriptors included in model (2) are listed in Supplementary File (Table S1). Results of internal validation of model (2) were presented in Table 6. Experimentally determined activities against *S. sclerotiorum* with the activities predicted by the best obtained QSAR models and residuals are given in Supplementary File (Table S3).

**Figure 3:**
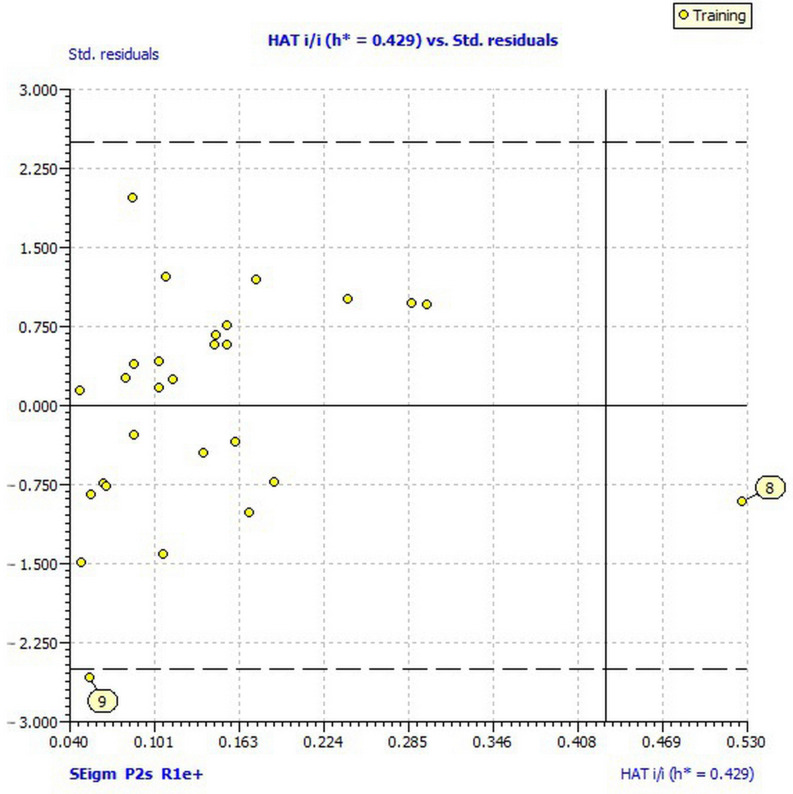
Williams plot of applicability domain of the QSAR model for inhibition of *Sclerotinia sclerotiorum* calculated by model (2).

The model satisfied the fitting and internal validation criteria, confirming the stability of the model: *R*^2^ and *R*^2^_adj_ ≥ 0.60; *CCC*_tr_ ≥ 0.80; *Q*^2^*_loo_* > 0.05; *RMSE_tr_* < *RMSE_cv_*. The robustness of the obtained QSAR model was affirmed by *R*^2^*_Y_scr__* and *Q*^2^*_Y_scr__* values < 0.2, as *R*^2^*_Y_scr__* < *Q*^2^*_Y_scr__* (Table 6). Also, the collinearity of descriptors in the model (2) was not detected since Δ*K* is higher than 0.05. Additionally, the absence of intercorrelation between the descriptors was verified by a low correlation coefficient in the matrix of intercorrelation (Table 7). Williams plots (Figure 3) reveal one outlier (compound **9**, with residual greater than 2.5 standard deviation units), and one compound outside of the applicability domain (**8**). The leverage of compound **8** is greater than the warning leverage (h* = 0.429); therefore, its estimated value must be interpreted with great care. By applying the derived model 2 on the previously excluded, low active compounds as members of the test set, according to external validation parameters, predictivity of the model failed (Table 6). This was expected because the compounds in the test set did not represent a chemical domain of the training set [30]. 

The first variable in model (2), *SEigm*, is a topological descriptor calculated by the eigenvalues of a square matrix representing a molecular graph. Descriptor *SEigm* presents an eigenvalue sum from a mass-weighted distance matrix [46]. Since *SEigm* makes a positive contribution to the activity, the presence of large substituents with heavy atoms (such as Br), implies increased inhibition of compound against *S*. *sclerotiorum*, as compound **8** (Table S2). The second variable *P2s* is the 2nd principal component shape directional WHIM index. This variable encodes relevant information about the 3D-distribution of the atomic electrotopological state (E-state). It encodes electronic and topological information about both heavy atoms and their bonded hydrogen. Since the more linear molecules have higher values of the P2s descriptor, the phenyl ring in the substituents reduces its value, and according to the negative coefficient *P2s* in the model (2), increases inhibition. For example, molecule **3**, which exhibited high inhibition (log % = 1.812), has 3-benzoyl and 7-benzyloxy groups (Table 1), therefore, the lowest value of *P2s* (Table S2). The electrotopological state of atoms depends on the number of π and lone pair electrons associated with skeletal atoms, which are expressed by intrinsic values (*I*) [46,49]. The influence of the electronic and topological state of the atom in the molecule on their inhibition effects can be explained by the comparison of two compounds, **6** and **7**. These two compounds differ in substituents at position C-6: compound **6** has hydroxyl group and compound **7** chlorine atom. Since -OH has a higher intrinsic value (6.00) than –Cl (4.111), compound **6** has a higher value of *P2s*, and therefore a weaker inhibition effect (Table 1 and Table 2, Table S2). The third variable, *R1e+*, is a descriptor of R maximal autocorrelation of lag 1, weighted by Sanderson electronegativity, which belongs to the GETAWAY (GEometry, Topology, and Atom-Weights AssemblY) descriptors [50]. The given descriptor is derived from the representation of the molecular structure called the influence/distance matrix, **R**, where the elements of the molecular influence matrix are combined with geometric interatomic distances in the molecule. The highest influences on the value of *R1e+* have the external atoms at the small interatomic space (at topological distance 1), taking into account their Sanderson electronegativity. Therefore, molecules with a higher number of terminal electronegative atoms (O, Cl, N) have higher values of *R2e*+ (Table S1) and enhanced inhibition. In summary, substituents that enhance inhibitory activity of coumarin derivative against *S*. *sclerotiorum* are: benzoyl groups, heavy atoms, such as Br, and groups with electronegative atoms –OH, OCH_3_, -Cl) at the specific position of the coumarin skeleton due to mutual sterical repulsion.

**Figure 4:**
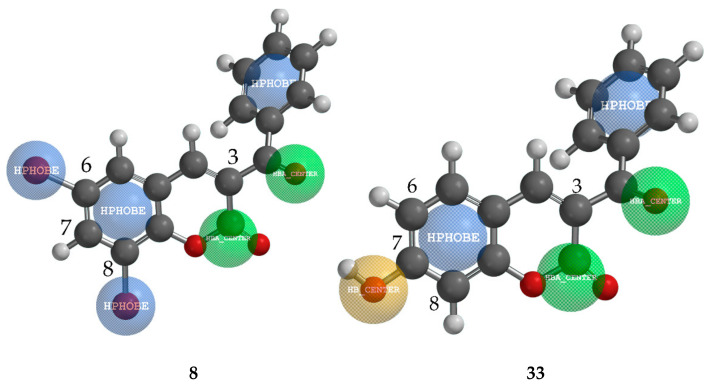
Pharmacophore mapping of one of the most active compounds **8** and one of the least active compound **33** against pathogen fungi *S. sclerotiorum*. (Green = hydrogen-bond acceptor; Blue = hydrophobe region; Brown = hydrogen-bond donor).

Pharmacophore mapping of two structurally similar compounds (**8** and **33**) with the opposite effect on *S*. *sclerotiorum* (84.70% and 6.83%, respectively) revealed the importance of the substituent at the position C-7 (Figure 4). Both compounds possess a hydrophobic benzoyl group at position C-3. While compound **8** has two hydrophobic Br atoms at positions C-6 and C-8, compound **33** has only an electron-donating group –OH at position C-7, which strongly acts against inhibition.

### 2.5. Molecular Docking Study

In order to determine the possible mechanism of action of coumarin derivatives against pathogenic fungi, a molecular docking study has been performed on three enzymes responsible for the fungal growth: demethylase (sterol 14α-demethylase (CYP51), pdb ID: 5eah) [24]; chitinase (pdb ID: 4txe) [51]; transferase (N-myristoyltransferase, pdb ID: 2p6g) [28]; and the three plant cell wall-degrading enzymes: endoglucanase I (pdb ID: 2ovw) [52]; proteinase K (pdb ID: 2pwb) [53]; pectinase (endopolygalacturonase, pdb ID: 1czf) [54]. The compounds were ranked by an energy-based scoring function. The docking scores of the first ten best-docked poses are presented in Table 8. In order to prove the specificity of the compounds, molecular docking was performed on the enzyme that is not related to fungal growth. For this purpose, we have chosen acetylcholinesterase (AChE), the target enzyme for nematocides (pdb ID: 1eve) [55].

Comparison of the results shown in Table 8 with the experimentally obtained antifungal activity (Table 2) of the analyzed compounds revealed that the best ten docking scores of molecular docking on transferase, proteinase K, and pectinase are in the best agreement with antifungal bioassay results against *S. sclerotiorum*. Thus, among the first ten compounds with the highest inhibition activity against *M. phaseolina*, compound **36** (79.01% inhibition) is among the first ten binding energies on all enzymes, except transferase. Similarly, compound **21** (74.97% inhibition *of M. phaseolina*) has high binding energies on endoglucanase and pectinase. The four compounds that achieved the ten best binding energies in complex with proteinase K (**6**, **5**, **1**, **8**) and pectinase (**15, 14, 6, 5**) are among the ten strongest inhibitors of *S. sclerotiorum*. Two compounds (**36** and **21**), whose inhibitions are among the top ten against *F. oxysporum* f. sp. *lycopersics*, showed binding affinity for pectinase only. No relation was observed between the inhibition activities against *F. culmorum* and the docking scores for all observed enzymes.

**Figure 5:**
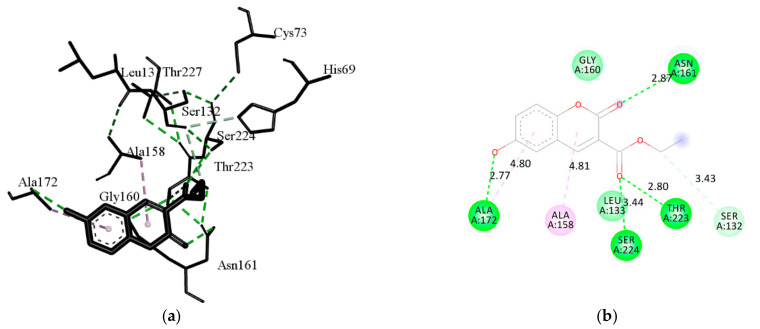
The main interactions of compound **6** with residues in the binding site of proteinase K: (**a**) 3D representation of the binding site; (**b**) 2D representation of main interactions with interatomic distances (Å). (green = conventional hydrogen bond; light green = van der Waals; very light green carbon-hydrogen bond; purple = π–alkyl interactions).

**Figure 6:**
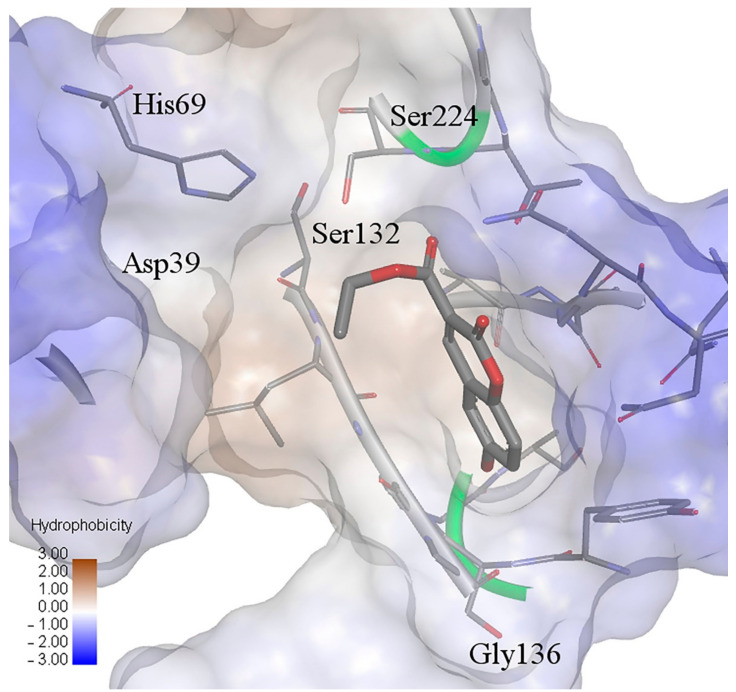
Hydrophobic surface representation of proteinase K active site with docked compound **6**.

Compound 3 proved to be the most promising ligand, making a complex with almost all enzymes (except proteinase K), but although this has not been experimentally determined, it is not surprising since this compound was estimated as highly toxic (Table 3). The results of docking on acetylcholinesterase have shown that compound **3** has also the lowest binding energy. In addition, compounds **8**, **33**, and **37**, with the first ten highest binding energies, have estimated the highest aquatic toxicity. According to the total energies that are in the range of binding energies of other enzymes related to the antifungal activities, the coumarins are also promising candidates for inhibition of pathogenic nematodes. Compound **6** has the lowest binding energy, therefore, it best fits into the active site of proteinase K, even better than the ligand from the original complex, coumarin. This compound exhibited good inhibition against growth of *M. phaseolina* (66.32%) and *S. sclerotiorum* (82.65%) (Table 2), and its possible mechanism is an inhibition enzyme responsible for cell-wall protein degradation. The energies of the interactions between the protein residues and ligand **6** in docked pose 0 are tabulated in Table 9. The binding site was defined according to the crystal structure of the complex coumarin with proteinase K (pdb ID: 2pwb). Figure 5 illustrates the interactions of compound **6** with residuals of the receptor, while Figure 6 shows a hydrophobic surface representation of the proteinase K binding site with docked compound **6**. Proteinase K belongs to the group of serine proteases, which hydrolyze the peptide bonds via a nucleophilic serine residue in the active site [56]. The active site of proteinase K consists of the catalytic triade Asp39…His69…Ser224, and substrate recognition site (Gly100-Tyr104, and Ser132-Gly136) [57]. Compound **6** forms four strong hydrogen bonds: oxygen atoms from the 6-OH group with Ala172; oxygen atoms from the 3-carbonyl group with Ser224 and Thr223, and oxygen atoms from the 2-keto group with Asn161. This confirms the results of the QSAR study about the importance of group with electronegative atoms from hydroxyl and acetyl groups for enhanced inhibitory effects of coumarin derivatives. A carbon-hydrogen bond is formed between ethyl groups of 3-COOCH_2_CH_3_ and Ser132. As well, the strongest van der Waals interaction forms with Gly134, Leu133, Gly160, Ala159, Gly135, and Asn161.

## 3. Discussion

The coumarin derivatives analyzed showed good activity against plant pathogenic fungi and were generally safe for beneficial bacteria and nematodes, making them potential candidates for environmentally friendly, plant-protection products. Taking into consideration all tested biological effects (antifungal, antibacterial, nematicidal, estimated toxicity) of analysed compounds, the most promising candidate is molecule **25**. This molecule has demonstrated optimal antifungal effects against all analysed pathogenic fungi and did not affect beneficial bacteria and nematodes (Table 2). As well, low toxicity for rats and aquatic organisms, low bioaccumulation, and non-mutagenicity were estimated for the same compound (Table 3). Compounds **28** and **29** demonstrated potential to become environmentally friendly, plant-protection compounds, with very good antifungal activity, (Table 2), but they were estimated as potentially mutagen (Table 3). Compound **7** demonstrated high inhibition activity and, specific only against *S. sclerotiorum* (84.02%, Table 2), not harmful for beneficial bacteria and nematodes and non-toxic.

All mentioned compounds (**7**, **25**, **28**, and **29**) have structural features (Table 1) that have been displayed as favorable for enhanced antifungal activities: molecules with linear substituents (-CN, -OCH_3_, -OC_2_H_5_), and with more heteroatoms higher values of Pauling electronegativity index and with –Br atoms. These results are consistent with previous results of the SAR study of antifungal activity of coumarin derivatives. A study by Araújo et al. [58] showed that the aliphatic chain or electron-withdrawing groups enhanced the antifungal activities of coumarins. Thus, a commercial botanical fungicide in China, osthenol, is a simple derivative of coumarin that possesses 7-hydroxyl and 8-prenyl groups. Prenylation at C-8 is related to the improved lipophilicity of osthenol, which favours its permeation through the lipid layer of the fungi [59]. Song et al. [8] stressed the importance of bromine as substituents for higher antifungal activity. A 3D-QSAR study of Wei et al. [9] relieved that small electron-withdrawing substituents of coumarin’s phenyl ring and hydrophilic electron-donating groups on the coumarin’s pyrone ring could enhance the antifungal activity. Moreover, 4-methyl coumarin with benzoyl group at the C-7 position has the only one that showed significant activity against *Fusarium solani* among the other compounds [60].

To elucidate the inhibitory mechanism of the tested coumarins against plant pathogenic fungi, the results most similar to the experimental ones were obtained by molecular docking to the binding site of enzymes that degrade plant cell walls, proteinases K, and pectinases. Plant pathogenic fungi secrete a wide range of cell-wall-degrading enzymes, such as glycanases and proteases, that are depolymerized cell wall components during the colonization of the host plant [27,61]. Since the plant cell walls possess several structural proteins, fungal proteases are important during the infection process and are key factors for fungal pathogenicity. Proteinases also play important roles in fungal nutrition [62]. The results of the molecular docking study for proteinase are in the best agreement with inhibition of *S. sclerotiorum*, since that necrotrophic fungus destroys plant tissues during infection by various enzymes, such as proteinases [63]. A large group of fungal proteolytic enzymes is serine proteases, named by the serine residue at the active site of the catalytic triad Ser-195, His-57, and Asp-192. Proteinase K is a serine proteinase that hydrolyses the peptide bonds via a nucleophilic serine residue in the active site. The mechanism of its catalysis consists of the acylation and the deacylation reactions [64,65].

Observed derivatives of compounds have been shown as potent antifungal agents mostly against *M. phaseolina* and *S. sclerotiorum*, but according to the results of bioassay, they were not harmful against beneficial bacteria and nematodes. To the best of our knowledge, this is the first report on the effects of coumarin derivates on EPNs. The biological activity of coumarins has been reported against plant-parasitic nematodes and rhabditid nematodes other than EPNs, causing more than 90% nematode mortality [66,67]. For instance, in mortality bioassay of furocoumarins extracted from parsley (*Petroselinum crispum*) against plant-parasitic nematodes (*Meloidogyne* spp.), xanthotoxol was found as the most active furanocoumarin, followed by psoralen, which lacks the hydroxyl group, and xanthotoxin, which has a methoxy group. Coumarins are plant constituents, and their derivates as a botanical nematicide have attracted considerable interest due to their favorable biorational profile [68]. The methods and reports of the relevant studies with *C. elegans* are not standardized, and effective concentrations of the nematode active compounds are often reported in different units [16]. The median lethal concentration (LC_50_) values of coumarins in previous studies depended on the tested compound. For instance, the LC_50_ of psoralen was found to be 119.40 mg L^−1^ against *C. elegans*, nematode closely related to EPNs, while the L_C50_ for the plant-parasitic nematodes was higher [69]. The differences in concentrations of active compounds found in previous studies could be related to different pharmacokinetics of specific groups of nematodes and molecular targets [70]. In our study, only compound 10 in concentration 500 mg L^−1^ caused more than 50% nematode mortality. Other tested compounds should be further bioassayed in real scenario systems to confirm the compatibility with beneficial soil nematodes.

The estimated toxicity of compounds has shown that some antifungal active compounds are potentially toxic against water organisms and rats, and these should be excluded from the design of future compounds. Although coumarins are naturally occurring substances, and their presence in food is usually safe for humans [71], some of their derivatives have a hepatotoxic effect on rats [72] and humans [73]. Also, warfarin, or 3-(α-acetonylbenzyl)-4-hydroxycoumarin, an oral anticoagulant drug, has shown teratogenicity and embryo lethality on zebrafish embryos [74]. Warfarin is also regarded as a potential pollutant in the aquatic environment [75]. Evaluation of toxic effects on *S. typhimurium* strains has shown that 6,7-hydroxycoumarins and 4-methylesculetin did not exert mutagenicity, but 4-methylesculetin induced greater cytotoxicity at high concentrations than 6,7-hydroxycoumarins [76]. Polyphenols, secondary plant metabolites that include phenolic acids, tannins, coumarins, lignins, stilbenes, terpenes, and flavonoids, naturally offer plants protection against abiotic stresses, UV light, pathogens, parasites, and plant predators [77]. Xanthohumol, a prenylated flavonoid isolated from hops (*Humulus lupulus* L.), was proven to have chemoprotective effects against the carcinogenic food contaminant aflatoxin B1 that is produced by the fungi *Aspergillus flavus* and *Aspergillus parasiticus* [78]. Its antimutagenic effect is based on preventing the DNA adduct formation and DNA damage induction.

New compounds, which structures are based on secondary plant metabolites, could be promising active components of environmentally and toxicologically acceptable plant protection products.

## 4. Materials and Methods

### 4.1. Biological Asay

#### 4.1.1. Antifungal Assay

For the preparation of stock solutions of compounds, a concentration of 4 μmol/mL corresponding mass was dissolved in 2.5 mL of DMSO and 2.5 mL of distilled water. The volume of 1 mL of stock solution was added to the mixture to produce the final compound concentration of 0.08 μmol/mL, and to keep the amount of DMSO in the mixture at 1%. As a control, untreated potato dextrose agar (PDA) was used. The antifungal assay was performed on 4 cultures of phytopathogenic fungi (*Fusarium oxysporum* f. sp. *lycopersici, Fusarium culmorum*, *Macrophomina phaseolina* and *Sclerotinia sclerotiourum*). The test was carried out according to the method of Siber et al. [79]. Petri dishes were kept in a growth chamber at 22 ± 1 °C, with a 12 h light/12 h dark regime. Each measurement consisted of four replicates. The radial growth of the fungal colonies was measured 48 h after inoculation. The in vitro inhibiting effects of the test compounds on the fungi were calculated by the antifungal index (% inhibition) [80].

#### 4.1.2. Antibacterial Activity

For antibacterial activity, a 5.12 mg/mL stock solution of each tested compound was prepared by dissolving 1.024 mg of the compound in 20 μL of DMSO and adding up to 200 μL of distilled water.

Antibacterial activity was determined by the broth microdilution method to obtain the minimum inhibitory concentration (MIC) of the tested bacteria. Compounds were diluted from 512 to 1 μg/mL. The tested bacteria included beneficial soil organisms Gram-negative bacteria *Bacillus mycoides* and Gram-positive bacteria *Bradhyrhizobium japonicum*. Bacterial cultures were multiplied on nutrient agar (Liofilchem, Italy) and Vincent agar [14], while the antibacterial activity was tested on the same broth media. All substances were dissolved in dimethyl-sulfoxide (DMSO) and transferred to a sterile 96-well microdilution plate with 50 μL of the appropriate medium. Plates were inoculated with inoculum according to methods described in Wiegand et al. [81]. The plates were incubated and the results were checked after 48 h. The experiment was set up in four replicates.

#### 4.1.3. Nematicidal Activity

For the preparation of the 500 μg/mL stock solutions, 2 mg of each compound was dissolved in 20 μL of DMSO and 3980 μL of distilled water containing 0.1% Triton. Inhibition of nematode motility and mortality was tested for all compounds in a maximum concentration of 500 μg/mL with four repetitions in a 24-well plate.

Entomopathogenic nematodes (EPNs), *Heterorhabditis bacteriophora* and *Steinernema feltiae* are affected by pesticides as non-target soil organisms. For the next generation of chemical PPP, compatibility with biological control agents is desirable; however, incompatible chemical compounds could be further tested against plant-parasitic nematodes and helminthic parasites of animals and humans. An aliquot of approximately 100 infective juveniles *H. bacteriophora* (indigenous Croatian strain ISO9, Gen-Bank accession numbers MG944244) was placed in each well containing 250 μL of the working solution. The same procedure was used for EPNs species *S. feltiae* (indigenous Croatian strain ISO16, GenBank accession numbers MG952287). Distilled water containing DMSO and Triton was used as a control. Well plates were incubated in the dark at 24 °C. The number of motile, dead, and live nematodes was recorded after 48 h. Nematodes were observed under a microscope (40× magnifications) and considered dead when they failed to respond to physical stimuli with a probe. The values were determined as percentage corrected mortality according to the Schnei-der-Orelli formula.

### 4.2. Computational Methods

#### 4.2.1. Calculation of Toxicity

The toxicity of compounds was calculated entering their SMILES notation into the program T.E.S.T. v.4.1 using two different QSAR methodologies: single model method and consensus method. The program provides estimated thresholds of toxicity based on the number of models that estimate toxicity thresholds by read-across among structural analogs or via multivariate regression [32].

#### 4.2.2. QSAR Methods

Antifungal activities of tested compounds, expressed as % inhibition, were converted in the form of the logarithm values (log % inhibition).

The 3D structures of coumarin derivatives were optimized by Spartan ’08 (Wavefunction, Inc.; Irvine, CA, USA, 2009), using the molecular mechanics force field (MM+) [82] and subsequently by the semiempirical AM1 method [83]. The molecular structures were optimized using the Polak-Ribiere algorithm until the root mean square gradient (RMS) was 0.001 kcal/mol per Å. A descriptors calculation was performed using Parameter Client (Virtual Computational Chemistry Laboratory, an electronic remote version of the Dragon program [84]. Employing QSARINS-Chem 2.2.1 (University of Insubria, Varese, Italy) [85], from the huge pool of calculated descriptors, the following were excluded: descriptors with a constant value for more than 80%, and descriptors that were too inter-correlated (>70%). The final number of descriptors selected for the generation of models was 1483.

The compounds for the test set were chosen using the joining tree clustering method based on the whole set of selected descriptors, including the activity, employing Statistica 7.0 (StatSoft, Inc.; Tulsa, OK, USA). As the distance measure, we used the Euclidean distance with the Single linkage as a linkage rule.

The best QSAR models were obtained by the Genetic Algorithm (GA) using QSARINS. Given the number of molecules in the data set (38) the number of descriptors (*I*) in the multiple regression equation was limited to three [86]. The models were assessed by: fitting criteria; internal cross-validation using the leave-one out (LOO) method; and external validation. The robustness of QSAR models was tested by the Y-randomisation test. Investigation of the applicability domain of the prediction model was performed by Williams plots (plotting residuals vs. leverage of training compounds) in order to identify the outliers and influential chemicals. The predicted data for chemicals with leverage values higher than the warning leverage (h*) must be considered with caution. The warning leverage h* is defined as 3*p*′/*n*, where n is the number of training compounds and *p*′ is the number of model parameters [87]. Pharmacophore mapping was performed using Spartan ’08, which may recognize six different chemical function descriptors (CFDs): hydrophobe; aromatic, hydrogen-bond donor, hydrogen-bond acceptor, positive and negative ionizable site.

#### 4.2.3. Molecular Docking

The molecular docking of compounds (**1**–**38**) was performed using iGEMDOCK (BioXGEM, Hsinchu, Taiwan). Crystal coordinates of six enzymes with co-crystalized standard inhibitor (sterol 14α-demethylase (CYP51), pdb ID: 5eah); chitinase, pdb ID: 4txe; N-myristoyltransferase, pdb ID: 2p6g; endoglucanase I, pdb ID: 2ovw; proteinase K, pdb ID: 2pwb; endopolygalacturonase, pdb ID: 1czf); were provided from Protein Data Bank (PDB, https://www.rcsb.org/, accessed on 14 May 2021)). Applying the generic evolutionary method, each compound was docked into the binding site using the following parameters: population size was 200, generations were 70 and the number of poses was 3. iGEMDOCK generates protein-compound interaction profiles based on electrostatic (E), hydrogen-bonding (H), and van der Waals (V) interactions. Compounds were ranked by combining the pharmacological interactions and energy-based scoring function: Total Energy = vdW + Hbond + Elec. Receptor-ligand interactions were visualized with BIOVIA Discovery Studio Visualizer 4.5 (Dassault Systèmes, San Diego, CA, USA).

## 5. Conclusions

Coumarin derivatives have shown different antifungal activities in vitro against four fungal plant pathogens. The most effective were against *M. phaseolina* and *S. sclerotiourum.* Generally, tested compounds were not harmful against soil-beneficial nematodes and bacteria. Compound **25**, which possesses 3-CN and 6-OH groups at the coumarin scaffold, has shown antifungal activities against all fungi tested, is nontoxic, and not harmful against beneficial bacteria and nematodes. A QSAR study showed that coumarin derivatives with multiple electron-withdrawal groups, especially at the position C-3, have enhanced activities against *M. phaseolina*, while coumarins with hydrophobic benzoyl groups at the position C-3, and –Br, –OH, OCH_3_, -Cl at the benzene ring of coumarin inhibit more strongly *S. sclerotiourum*. A possible mechanism of action of the tested compounds is their inhibitory effect against plant wall-degrading enzymes. Analyzed coumarin derivatives are promising candidates for developing plant-protection products that could be safe for the environment, human health, and non-target organisms.

## Figures and Tables

**Table 1:** Structures of analyzed compounds.

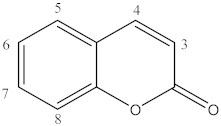		
No. mol	Mol ID	Substituents
**1**	**A3**	3-COOC_2_H_5_
**2**	**A5**	3-COC_6_H_5_
**3**	**C5**	3-COC_6_H_5_; 7-OCH_2_C_6_H_5_
**4**	**G3**	3-COOC_2_H_5;_ 6-Br
**5**	**J2**	3-COOCH_3_; 6-OH
**6**	**J3**	3-COOC_2_H_5_; 6-OH
**7**	**K3**	3-COOC_2_H_5_; 6-Cl
**8**	**L5**	3-COC_6_H_5_; 6-Br; 8-Br
**9**	**M2**	3-COOCH_3_; 7-OCH_3_
**10**	**M3**	3-COOC_2_H_5_; 7-OCH_3_
**11**	**N2**	3-COOCH_3_; 6-OCH_3_
**12**	**O3**	3-COOC_2_H_5_; 8-OC_2_H_5_
**13**	**O5**	3-COC_6_H_5_; 8-OC_2_H_5_
**14**	**P2**	3-COOCH_3_; 6-N^+^OO^−^
**15**	**P3**	3-COOC_2_H_5_; 6-N^+^OO^−^
**16**	**A1**	3-COCH_3_
**17**	**A4**	3-CN
**18**	**D1**	3-COCH_3_; 8-OH
**19**	**D4**	3-CN; 8-OH
**20**	**E1**	3-COCH_3_; 7-OH
**21**	**F1**	3-COCH_3_; 7-N(C_2_H_5_)_2_
**22**	**G1**	3-COCH_3_; 6-Br
**23**	**G4**	3-CN; 6-Br
**24**	**J1**	3-COCH_3_; 6-OH
**25**	**J4**	3-CN; 6-OH
**26**	**M4**	3-CN; 7-OCH_3_
**27**	**N4**	3-CN; 6-OCH_3_
**28**	**O1**	3-COCH_3_; 8-OC_2_H_5_
**29**	**O4**	3-CN; 8-OC_2_H_5_
**30**	**A2**	3-COOCH_3_
**31**	**C4**	3-CN; 7-OCH_2_C_6_H_5_
**32**	**E2**	3-COOCH_3_; 7-OH
**33**	**E5**	3-COC_6_H_5_; 7-OH
**34**	**G2**	3-COOCH_3_; 6-Br
**35**	**K5**	3-COC_6_H_5_; 6-Cl
**36**	**M5**	3-COC_6_H_5_; 7-OCH_3_
**37**	**J5**	3-COC_6_H_5_; 6-OH
**38**	**L3**	3-COOC_2_H_5_; 6-Br; 8-Br

**Table 2:** Results of antifungal, antibacterial and nematicidal activity of 38 coumarin derivatives. (^a^ inhibition rate %, 48 h after inoculation at the concentration 0.08 μmol/mL; ^b^ minimum inhibitory concentration (MIC/μg mL^−1^); ^c^ percentage corrected mortality, %, 48 h after inoculation at the concentration 500 μg/mL).

	Antifungal Activity ^a^				Antibacterial Activity ^b^		Nematicidal Activity ^c^	
No. mol	*Macrophomina phaseolina*	*Sclerotinia sclerotiorum*	*Fusarium oxysporum* f. sp. *lycopersici*	*F. culmorum*	*Bacillus mycoides*	*Bradhrizobium japonicum*	*Heterorhabditis bacteriophora*	*Steinernema feltiae*
**1**	53.63	73.09	−3.64	−9.53	>512	>512	8.00	10.00
**2**	57.67	39.62	1.21	−8.67	>512	>512	29.75	18.75
**3**	53.63	64.89	4.85	−7.80	>512	>512	0.00	0.00
**4**	56.52	77.87	1.21	−0.87	>512	>512	0.00	3.75
**5**	64.01	76.50	−6.07	−6.07	>512	64	0.00	0.00
**6**	66.32	82.65	14.56	26.86	>512	>512	31.25	20.75
**7**	65.74	84.02	10.92	13.86	>512	512	0.00	0.00
**8**	66.32	84.70	13.35	6.93	>512	>512	0.00	0.00
**9**	74.39	84.70	13.35	10.40	>512	512	0.00	0.00
**10**	72.09	64.89	19.42	13.86	>512	>512	56.75	64.00
**11**	53.06	51.91	6.07	−6.93	>512	>512	0.00	4.25
**12**	69.78	59.43	−7.28	−27.73	>512	>512	24.75	21.00
**13**	24.80	67.62	−6.07	−32.93	>512	>512	0.00	0.00
**14**	55.94	81.97	−10.92	−4.33	>512	512	0.00	0.00
**15**	59.98	76.50	0.00	−6.93	>512	512	0.00	2.00
**16**	61.71	43.72	27.91	30.33	>512	>512	0.00	0.00
**17**	61.71	38.93	23.06	26.00	>512	>512	8.75	14.75
**18**	59.98	27.32	27.91	9.53	>512	>512	0.00	0.00
**19**	69.78	54.64	32.77	13.00	512	>512	20.75	11.25
**20**	65.74	4.78	29.13	−3.47	512	>512	5.50	17.00
**21**	74.97	40.98	40.05	26.00	>512	>512	13.00	22.50
**22**	70.36	43.72	29.13	33.80	>512	>512	0.00	0.00
**23**	83.62	−15.03	27.91	−9.53	>512	>512	0.00	2.75
**24**	80.16	55.33	64.32	70.19	>512	>512	44.50	43.00
**25**	74.39	54.64	66.75	62.39	>512	>512	0.00	1.25
**26**	72.66	23.91	40.05	45.06	512	>512	12.00	14.25
**27**	75.55	61.48	71.60	63.26	512	512	0.00	0.00
**28**	76.12	79.92	65.53	65.86	>512	>512	0.00	0.00
**29**	77.28	66.26	65.53	70.19	>512	>512	0.00	0.00
**30**	70.93	−7.51	20.63	−17.33	>512	>512	0.00	0.00
**31**	69.20	0.68	32.77	−1.73	>512	>512	0.00	0.00
**32**	79.58	−4.10	24.27	−11.27	>512	>512	17.50	10.50
**33**	68.63	6.83	27.91	−10.40	>512	>512	0.00	10.50
**34**	66.90	−15.71	27.91	−10.40	>512	>512	16.50	40.25
**35**	78.43	−26.64	35.19	6.93	>512	>512	0.00	0.00
**36**	79.01	51.91	44.90	23.40	>512	>512	0.00	9.00
**37**	64.01	13.66	31.55	0.00	>512	>512	0.00	4.75
**38**	80.16	10.25	35.19	1.73	>512	>512	0.00	0.00
**control**	0	0	0	0	0	0	0	0

**Table 3:** Estimated toxicity for 38 coumarin derivatives.

No. mol	Oral Rat LD_50_ (mg/kg bw) ^a^	*Tetrahymena pyriformis* pIGC_50_ 48-h (mol/L) ^b^	Fathead Minnow pLC_50_ 96-h (mol/L) ^c^	Mutagenicity Value (Result) ^d^	Bioaccumulation (logBAF/L kg^−1^) ^e^
**1**	978.2	4.03	4.59	0.45 (neg)	0.85
**2**	386.97	4.97	5.68	0.43 (neg)	1.52
**3**	147.37	5.86	7.12	0.18 (neg)	1.48
**4**	2223.31	4.66	5.24	0.14 (neg)	1.14
**5**	1146.8	4.37	4.43	0.28 (neg)	0.79
**6**	1592.38	4.15	4.78	0.36 (neg)	0.76
**7**	1251.57	4.54	5.08	0.26 (neg)	1.31
**8**	634.37	5.87	6.82	0.35 (neg)	2.09
**9**	1882.79	3.96	4.29	0.49 (neg)	0.68
**10**	2471.53	4.37	4.66	0.45 (neg)	0.77
**11**	975.12	3.74	4.52	0.44 (neg)	1.03
**12**	2358.27	4.14	5.07	0.45 (neg)	1.12
**13**	387.72	4.74	5.64	0.40 (neg)	1.42
**14**	1799.19	4.18	4.93	0.63 (pos)	0.45
**15**	1169.36	4.78	5.20	0.61 (pos)	0.48
**16**	774.04	5.01	4.27	0.03 (neg)	0.90
**17**	519.66	4.37	3.74	0.36 (neg)	1.24
**18**	1163.28	4.96	3.90	0.02 (neg)	0.78
**19**	313.76	4.37	3.71	0.34 (neg)	1.17
**20**	1212.38	5.10	4.17	0.03 (neg)	0.61
**21**	1882.68	4.96	4.87	0.75 (pos)	1.03
**22**	2307.19	5.11	4.63	0.23 (neg)	1.14
**23**	1733.09	4.14	4.20	0.30 (neg)	1.51
**24**	725.86	4.99	4.10	0.01 (neg)	0.79
**25**	418.02	4.31	3.90	0.37 (neg)	1.21
**26**	1097.06	4.2	3.96	0.47 (neg)	0.98
**27**	718.52	3.78	3.74	0.35 (neg)	1.29
**28**	1187.42	4.21	4.83	0.57 (pos)	1.06
**29**	320.31	4.12	3.69	0.50 (pos)	1.22
**30**	695.39	3.87	4.43	0.46 (neg)	0.83
**31**	190.96	4.41	5.60	0.61 (pos)	1.61
**32**	1515.42	4.15	4.63	0.33 (neg)	0.49
**33**	190.83	5.21	5.43	0.43 (neg)	1.32
**34**	2158.86	4.85	5.31	0.08 (neg)	1.18
**35**	100.83	5.27	5.87	0.54 (pos)	1.92
**36**	451.79	4.91	5.34	0.39 (neg)	1.29
**37**	139.64	5.00	5.68	0.57 (pos)	1.43
**38**	749.5	5.17	6.34	0.49 (neg)	0.85

^a^ mg of compound per bodyweight of the rat required to kill half of a tested population; ^b^ negative logarithm (pIGC_50_) of the concentration (mol/L) of compound in water that causes 50% growth inhibition to *Tetrahymena pyriformis* after 48 h; ^c^ negative logarithm (pLC_50_) of the concentration (mol/L) of compound in water that kills half of fathead minnows (*Pimephales promelas*) in 96 h; ^d^ estimates mutagenicity of compound on *Salmonella typhimuriu*; ^e^ logarithmic value of ratio of the concentration of compound in the tissue of an aquatic organism to its concentration in water (in litres per kilogram of tissue).

**Table 4:** The statistical results of QSAR models (1).

Statistical Parameters	Model 1
*N*_tr_	23
*N*_ex_	9
*R* ^2^	0.78
*R* ^2^_adj_	0.75
*s*	0.03
*F*	23.46
*Kxx*	0.20
Δ*K*	0.12
*RMSE_tr_*	0.02
*MAE_tr_*	0.02
*CCC_tr_*	0.88
*Q* ^2^ *_LOO_*	0.67
*RMSE_cv_*	0.03
*MAE_cv_*	0.02
*CCC_cv_*	0.82
*R* ^2^ *_Y_scr__*	0.14
*Q* ^2^ *_Y_scr__*	−0.28
*RMSE_AV Y_scr__*	0.09
*RMSE_ext_*	0.03
*MAE_ext_*	0.02
*R* ^2^ *_ext_*	0.67
*CCC_ext_*	0.81
*Q* ^2^ *_F_*_1_	0.62
*Q* ^2^ *_F_*_2_	0.61
*Q* ^2^ *_F_*_3_	0.68
*r*^2^*_m_* average	0.54
*r*^2^*_m_* difference	0.02
Applicability domain	h* = 0.522
*N* compounds outlier	-
*N* compounds out of app.dom.	-

LOO (the leave-one out); *R*^2^ (coefficient of determination); *R*^2^_adj_ (adjusted coefficient of determination); *s* (standard deviation of regression); *F* (Fisher ratio); *Kxx* (multivariate correlation index); Δ*K* (global correlation among descriptors); *RMSE_tr_* (root-mean-square error of the training set); *MAE_tr_* (mean absolute error of the training set); *CCC_tr_* (concordance correlation coefficient of the training set); *Q*^2^*_LOO_* (cross-validated explained variance); *RMSE_cv_* (root-mean-square error of the training set determined through the cross validated method; *MAE_cv_* (mean absolute error of the internal validation set); *CCC_cv_* (concordance correlation coefficient test set using cross validation); *R*^2^*_Y_scr__* (Y-scramble correlation coefficients); *Q*^2^*_Y_scr__* (Y-scramble cross-validation coefficients); *RMSE_AV Y_scr__* (root-mean-square error of Y-randomization); *RMSE_ex_* (root-mean-square error of the external validation set); *MAE_ex_* (mean absolute error of the external validation set); *R*^2^*_ext_* (coefficient of determination of validation set); *Q*^2^*_F_*_1_, *Q*^2^*_F_*_2_, *Q*^2^*_F_*_3_ (predictive squared correlation coefficients); *CCC_ext_* (concordance correlation coefficient of the test set); *r*^2^*_m_* average (average value of squared correlation coefficients between the observed and (leave-one-out) predicted values of the compounds with and without intercept); *r*^2^*_m_* difference (absolute difference between the observed and leave-one-out predicted values of the compounds with and without intercept); h* (warning leverage for the applicability domain of the model).

**Table 5:** Correlation matrix (correlation coefficient, *R*) for the descriptors included in model (1).

	*JGI6*	*Mor28v*	*L2e*
*JGI6*	1.00		
*Mor28v*	−0.16	1.00	
*L2e*	0.29	−0.22	1.00

**Table 6:** The statistical results of QSAR model (2).

Statistical Parameters	Model 2	Model 2a *
*N*_tr_	28	28
*N*_ex_		10 **
*R* ^2^	0.78	0.78
*R* ^2^_adj_	0.75	0.75
*s*	0.07	0.07
*F*	28.84	28.84
*Kxx*	0.22	0.22
Δ*K*	0.17	0.17
*RMSE_tr_*	0.07	0.07
*MAE_tr_*	0.06	0.06
*CCC_tr_*	0.88	0.88
*Q* ^2^ *_LOO_*	0.71	0.71
*RMSE_cv_*	0.08	0.08
*MAE_cv_*	0.07	0.07
*CCC_cv_*	0.84	0.84
*R* ^2^ *_Y_scr__*	0.11	0.11
*Q* ^2^ *_Y_scr__*	−0.24	−0.24
*RMSE_AV Y_scr__*	0.14	0.14
*RMSE_ext_*		1.51
*MAE_ext_*		1.44
*R* ^2^ *_ext_*		0.03
*CCC_ext_*		0.01
*Q* ^2^_*F*1_		−0.03
*Q* ^2^_*F*2_		−9.06
*Q* ^2^_*F*3_		−102.96
*r*^2^*_m_* average		−0.21
*r*^2^*_m_* difference		0.47
Applicability domain	h* = 0.429	
*N* compounds outlier	1 (**9**)	10 (**20**, **23**, **30**–**35**, **37**, **38**)
*N* compounds out of app.dom.	1 (**8**)	2 (**8**, **38**)

* model (2) applied to a test set containing previously excluded 10 low-active compounds (**).

**Table 7:** Correlation matrix (correlation coefficient, *R*) for the descriptors included in model (2).

	*SEigm*	*P2s*	*R1e+*
*SEigm*	1.00		
*P2s*	0.20	1.00	
*R1e+*	0.08	0.53	1.00

**Table 8:** Docking score energies (Total E/kcal mol^−1^) of interactions of the best ten docked poses of coumarin derivatives, including standard ligands * in complex with: demethylase (sterol 14α-demethylase (CYP51), pdb ID: 5eah); chitinase (pdb ID: 4txe); transferase (N-myristoyltransferase, pdb ID: 2p6g); endoglucanase I (pdb ID: 2ovw); proteinase K (pdb ID: 2pwb); pectinase (endopolygalacturonase, pdb ID: 1czf); AChE (acetylcholinesterase, pdb ID: 1eve.).

Demethylase		Chitinase		Transferase		Endoglucanase I		Proteinase K		Pectinase		AChE	
Comp. (Pose)	Total E	Comp. (Pose)	Total E	Comp. (Pose)	Total E	Comp. (Pose)	Total E	Comp. (Pose)	Total E	Comp. (Pose)	Total E	Comp. (Pose)	Total E
**3** (2)	−100.41	**3** (1)	−122.83	**3** (2)	−99.82	**3** (1)	−127.50	**6** (0)	−114.21	**3** (1)	−96.65	**3** (0)	−118.95
**5lw** * (1)	−96.19	**37** (2)	−121.58	**15** (1)	−93.73	**31** (1)	−123.92	**13** (1)	−114.07	**15** (0)	−89.25	**13** (0)	−112.30
**33** (0)	−86.71	**13** (0)	−115.99	**3lp** * (2)	−91.27	**15** (1)	−113.50	**5** (1)	−110.68	**14** (2)	−86.21	**31** (2)	−107.19
**31** (1)	−86.05	**33** (2)	−115.37	**14** (0)	−88.30	**13** (1)	−107.84	**36** (2)	−109.91	**31** (2)	−84.93	**8** (1)	−106.67
**13** (0)	−85.79	**38f** * (0)	−112.76	**13** (1)	−85.82	**14** (1)	−105.32	**37** (1)	−109.92	**13** (1)	−84.49	**33** (0)	−105.41
**15** (0)	−84.84	**6** (0)	−112.29	**37** (1)	−84.52	**36** (0)	−104.68	**33** (1)	−107.92	**6** (0)	−84.14	**15** (1)	−104.48
**37** (1)	−84.18	**36** (0)	−110.99	**31** (0)	−84.51	**12** (2)	−104.19	**2** (1)	−104.19	**36** (2)	−81.44	**36** (1)	−104.32
**36** (1)	−81.35	**8** (1)	−110.43	**12** (2)	−83.12	**21** (2)	−104.11	**1** (1)	−104.13	**21** (1)	−81.19	**37** (1)	−103.03
**8** (0)	−81.14	**31** (0)	−109.87	**35** (1)	−82.71	**33** (2)	−104.07	**12** (2)	−103.34	**33** (0)	−80.92	**12** (1)	−102.67
**14** (0)	−80.10	**5** (1)	−107.96	**8** (1)	−82.32	**6** (0)	−102.75	**8** (2)	−102.34	**5** (1)	−80.27	**2** (1)	−101.18

* pdb ID of standard ligand.

**Table 9:** The energies of the main interactions between proteinase K residues and compound **6**.

H Bond	Energy	Van der Waals Interaction	Energy
M-GLY-160	−3.76	S-HIS-69	−1.28
S-ASN-161	−9.68	M-SER-132	−3.58
M-SER-170	−5.61	M-LEU-133	−8.08
M-PRO-171	−3.50	M-GLY-134	−10.23
M-ALA-172	−3.50	M-GLY-135	−4.94
S-THR-223	−2.50	M-ALA-158	−4.13
M-SER-224	−3.50	M-ALA-159	−6.16
S-SER-224	−4.71	M-GLY-160	−7.28
		M-ASN-161	−3.49
		S-ASN-161	−4.87
		S-ASN-162	−2.68
		S-TYR-169	−2.09
		M-SER-170	−0.14
		M-SER-224	−1.72

(M = main chain; S = side chain).

## Data Availability

Data are contained within the article.

## Supplementary Materials

The following are available online at https://www.mdpi.com/article/10.3390/ijms22147283/s1, Figure S1. Dendrogram of a cluster formation for the activities against *M. phaseolina*.; Figure S2. Dendrogram of a cluster formation for the activities against *S. sclerotiorum*.; Table S1. Values of molecular descriptors included in model (1) and (2); Table S2. Experimental determined activities against *M. phaseolina* with the activities predicted by the best obtained QSAR model (1) and residuals; Table S3. Experimental determined activities against *S. sclerotiorum* with the activities predicted by the best obtained QSAR models and residuals.
