# Supplementary material for: Biological Activities Related to Plant Protection and Environmental Effects of Coumarin Derivatives: QSAR and Molecular Docking Studies
Source: Int J Mol Sci. 2021 Jul 6;22(14):7283. doi: 10.3390/ijms22147283 (PMC8303553; doi:10.3390/ijms22147283)
Supplement: Supplementary file 1 [file ijms-22-07283-s001.zip › ijms-1264012-supplementary.pdf]

International Journal of
Molecular Sciences

Supplementary Materials

Figure S1. Dendrogram of a cluster formation for the activities against *M. phaseolina*.

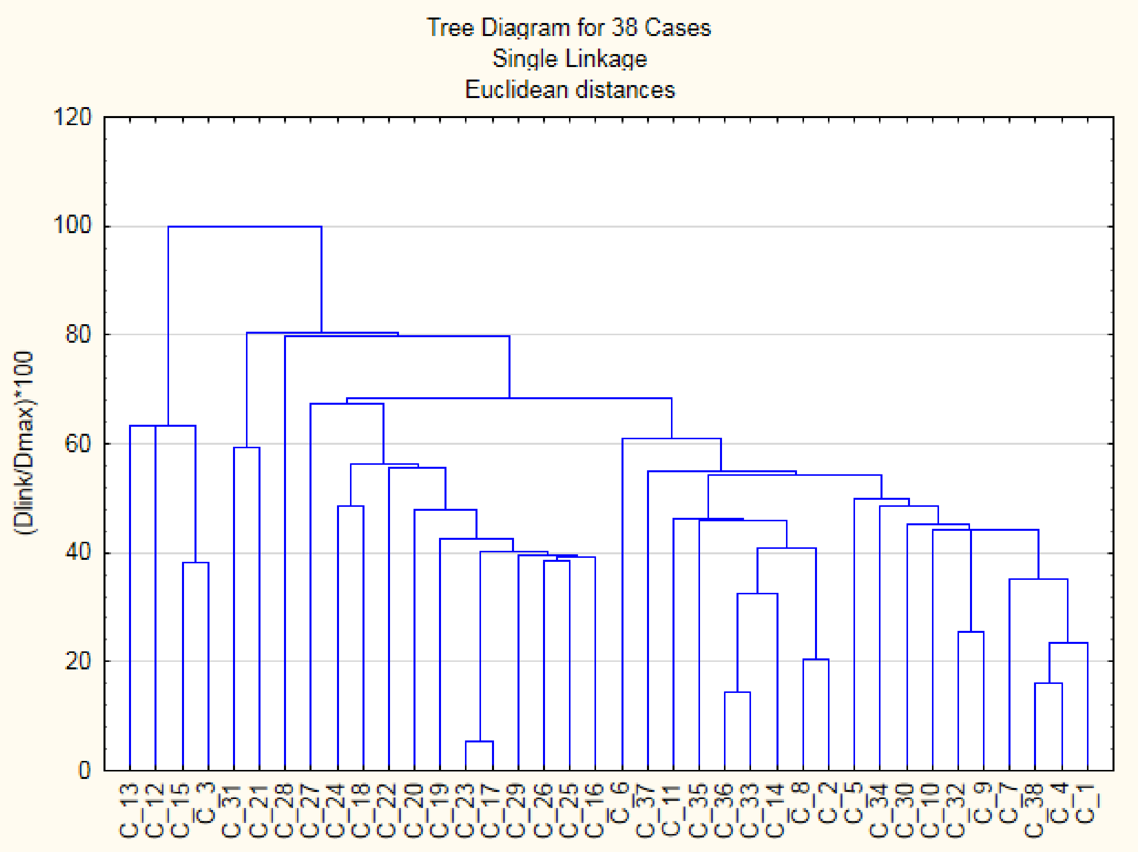

Table S1. Values of molecular descriptors included in model (1) and (2).

Molecule	log % inhibition *M. phaseolina*	*JGI6*	*Mor28v*	*L2e*	log % inhibition *S. sclerotiorum*	*SEigm*	*P2s*	*R1e+*
**1**	1.729	0.017	0.081	1.836	1.864	0.997	0.187	0.187
**2**	1.761	0.017	0.075	1.813	1.598	0.748	0.200	0.145
**3**	1.729	0.015	0.111	1.885	1.812	0.997	0.073	0.125
**4**	1.752	0.027	0.075	1.952	1.891	1.847	0.177	0.184
**5**	1.806	0.032	0.091	1.924	1.884	1.247	0.193	0.203
**6**	1.822	0.027	0.085	1.96	1.917	1.247	0.174	0.197
**7**	1.818	0.027	0.146	1.833	1.924	1.659	0.171	0.194
**8**	1.822	0.022	0.041	2.063	1.928	2.447	0.201	0.14
**9**	1.872	0.025	-0.023	2.089	1.928	1.247	0.182	0.158
**10**	1.858	0.022	-0.013	1.687	1.812	1.247	0.138	0.155
**11**	1.725	0.029	0.011	2.523	1.715	1.247	0.198	0.167
**12**	1.844	0.024	0.001	2.692	1.774	1.247	0.198	0.162
**13**	1.394	0.02	0.119	2.382	1.83	0.997	0.146	0.139
**14**	1.748	0.026	0.091	3.093	1.914	1.639	0.189	0.217
**15**	1.778	0.023	0.048	3.038	1.884	1.639	0.201	0.217
**16**	1.79	0.02	0.041	2.383	1.641	0.748	0.261	0.204
**17**	1.79	0.025	0.06	2.146	1.59	0.641	0.204	0.177
**18**	1.778	0.029	0.031	2.547	1.436	0.997	0.305	0.198
**19**	1.844	0.028	0.067	2.508	1.738	0.891	0.253	0.231
**20**	1.818	0.025	0.095	1.933	0.68	0.997	0.194	0.193
**21**	1.847	0.025	-0.005	2.477	1.641	0.891	0.127	0.126
**22**	1.904	0.036	0.014	2.528	1.743	1.598	0.223	0.204
**23**	1.922	0.037	0.036	2.232	0	1.491	0.19	0.175
**24**	1.872	0.036	0.047	2.494	1.738	0.997	0.209	0.208
**25**	1.878	0.037	0.057	2.136	1.789	0.891	0.18	0.206
**26**	1.881	0.03	0.01	1.843	1.903	0.891	0.136	0.195
**27**	1.888	0.03	0.042	1.962	1.821	0.891	0.143	0.199
**28**	1.829	0.029	0.013	2.974	1.426	0.997	0.243	0.138
**29**	1.829	0.028	0.052	3.214	1.468	0.891	0.212	0.162
**30**	1.851	0.02	0.025	2.281	0	0.997	0.269	0.213
**31**	1.84	0.015	-0.01	2.185	-0.166	0.891	0.068	0.125
**32**	1.901	0.024	0.027	2.193	0	1.247	0.21	0.211
**33**	1.836	0.019	0.073	1.806	0.834	0.997	0.163	0.16
**34**	1.825	0.032	0.012	2.443	0	1.847	0.24	0.213
**35**	1.894	0.021	0.141	1.939	0	1.409	0.194	0.14
**36**	1.898	0.02	-0.017	1.927	1.715	0.997	0.166	0.144
**37**	1.806	0.021	0.046	2.144	1.135	0.997	0.209	0.225
**38**	1.904	0.03	-0.032	2.456	1.011	2.697	0.262	0.186

Table S2. Experimental determined activities against *M. phaseolina* with the activities predicted by the best obtained QSAR model (1) and residuals

ID	Status	Exp. endpoint	Pred. by model eq.	Pred.Mod.Eq.Res.
1	Training	1.729	1.778	0.049
2	Prediction	1.761	1.783	0.022
3	Training	1.729	1.743	0.014
4	Excluded			
5	Prediction	1.806	1.850	0.044
6	Training	1.822	1.825	0.003
7	Training	1.818	1.787	-0.031
8	Training	1.822	1.824	0.002
9	Training	1.872	1.885	0.013
10	Prediction	1.858	1.880	0.022
11	Excluded			
12	Training	1.844	1.835	-0.009
13	Excluded			
14	Training	1.748	1.764	0.016
15	Prediction	1.778	1.780	0.002
16	Training	1.790	1.798	0.008
17	Prediction	1.790	1.823	0.033
18	Excluded			
19	Training	1.844	1.818	-0.026
20	Training	1.818	1.808	-0.010
21	Training	1.847	1.855	0.008
22	Prediction	1.904	1.900	-0.004
23	Training	1.922	1.903	-0.019
24	Training	1.872	1.878	0.006
25	Training	1.878	1.892	0.014
26	Prediction	1.881	1.900	0.019
27	Training	1.888	1.872	-0.016
28	Training	1.829	1.841	0.012
29	Training	1.829	1.797	-0.032
30	Prediction	1.851	1.814	-0.037
31	Training	1.840	1.816	-0.024
32	Excluded			
33	Prediction	1.836	1.796	-0.040
34	Training	1.825	1.883	0.058
35	Excluded			
36	Training	1.898	1.860	-0.038
37	Training	1.806	1.811	0.005
38	Training	1.904	1.903	-0.001

Figure S2. Dendrogram of a cluster formation for the activities against *S. sclerotiorum*.

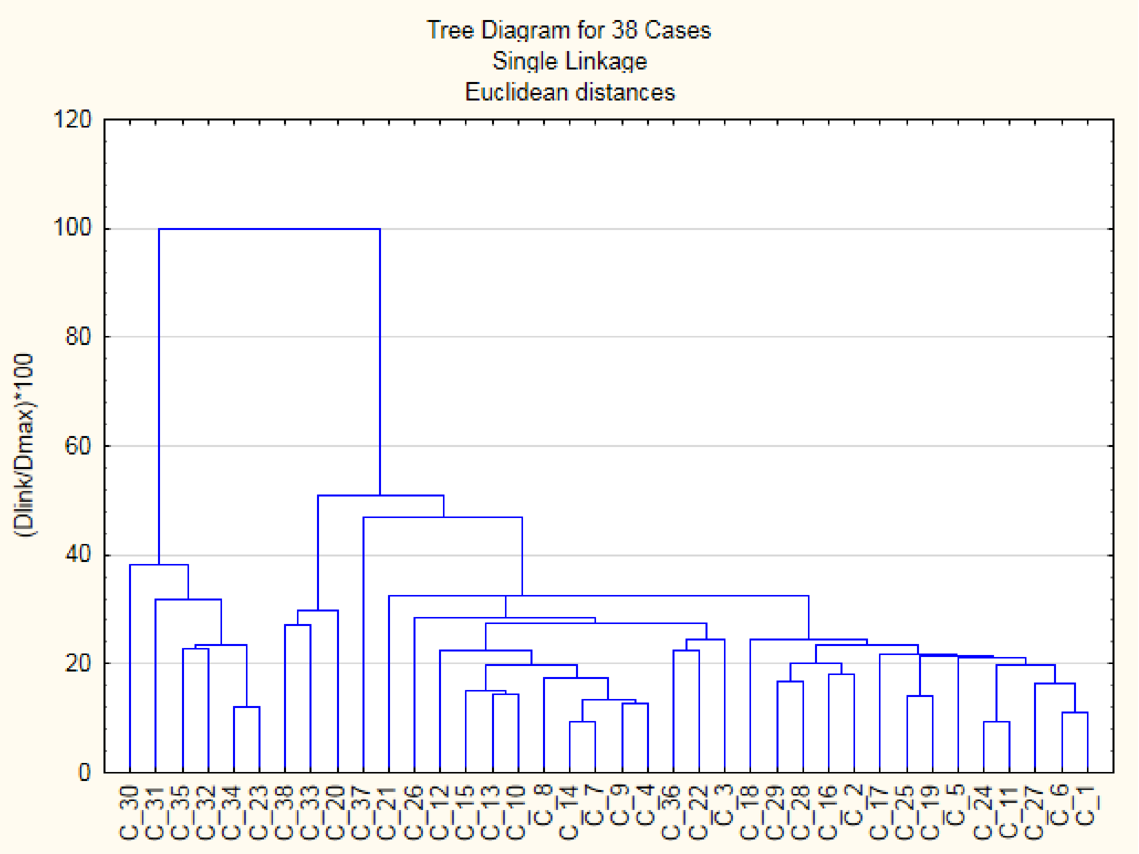

Table S3. Experimental determined activities against *S. sclerotiorum* with the activities predicted by the best obtained QSAR models and residuals

ID	Status	Exp. endpoint	Pred. by model eq.	Pred.Mod.Eq.Res.
1	Training	1.864	1.756	-0.108
2	Training	1.598	1.567	-0.031
3	Training	1.812	1.872	0.060
4	Training	1.891	1.938	0.047
5	Training	1.884	1.832	-0.052
6	Training	1.917	1.862	-0.055
7	Training	1.924	1.942	0.018
8	Training	1.928	1.882	-0.047
9	Training	1.928	1.742	-0.187
10	Training	1.812	1.841	0.029
11	Training	1.715	1.726	0.011
12	Training	1.774	1.713	-0.061
13	Training	1.830	1.731	-0.099
14	Training	1.914	1.954	0.040
15	Training	1.884	1.925	0.041
16	Training	1.641	1.572	-0.069
17	Training	1.590	1.620	0.030
18	Training	1.436	1.498	0.062
19	Training	1.738	1.690	-0.049
20	Excluded			
21	Training	1.641	1.723	0.082
22	Training	1.743	1.830	0.087
23	Excluded			
24	Training	1.738	1.757	0.019
25	Training	1.789	1.802	0.013
26	Training	1.903	1.880	-0.023
27	Training	1.821	1.874	0.053
28	Training	1.426	1.493	0.067
29	Training	1.468	1.610	0.142
30	Excluded			
31	Excluded			
32	Excluded			
33	Excluded			
34	Excluded			
35	Excluded			
36	Training	1.715	1.696	-0.020
37	Excluded			
38	Excluded			
